# Supplementary material for: Mapping the Environmental Co-Benefits of Reducing Low-Value Care: A Scoping Review and Bibliometric Analysis
Source: Int J Environ Res Public Health. 2024 Jun 22;21(7):818. doi: 10.3390/ijerph21070818 (PMC11276457; doi:10.3390/ijerph21070818)
Supplement: Supplementary file 1 [file ijerph-21-00818-s001.zip › Supp File 2_PRISBA Checklist.pdf]

Mapping the environmental co-benefits of reducing low-value care: A scoping review and bibliometric analysis
Parker et al., 2024

Supplementary File 2. Preferred Reporting Items for Systematic Bibliometric Analysis (PRISBA) Checklist.

SECTION		ITEM	PRISBA CHECKLIST ITEM	REPORTED ON PAGE #
**TITLE**				
Title		1	Identify the study as a bibliometric analysis within the title.	1
**ABSTRACT**				
Structured Summary		2	Provide a structured summary that includes: study background; study objectives; publication eligibility criteria; bibliometric metadata sources; methods related to data collection, data pre-processing, data analysis, and data visualization; results; discussion (interpretation of results); and conclusions that relate to the study objectives and research questions.	1 – 2
**INTRODUCTION / BACKGROUND**				
Rationale		3	Describe the rationale for conducting the bibliometric analysis in the context of what is already known about the research area of interest. Explain why the study objectives and research questions lend themselves to a bibliometric analysis approach.	4
Objectives & Research Questions		4	Provide an explicit statement outlining the study objectives and research questions being addressed	4
**METHODS**				
**Selection of publications for analysis**	Database(s) used	6	Identify and provide rationale for all databases used in the search. Identify the date the most recent search was executed.	1, 4, 5
	Publication eligibility criteria	5	Specify the publication eligibility criteria used (e.g., publication-type, years considered, language and status), and provide rationale.	5, Figure 1.
	Search String	7	Provide the search string(s) used. Also present (as additional file, if necessary) the full electronic search strategy for at least one database, including any limits used, such that it could be repeated.	5
	Selection of publications	8	State the process for selecting publications (e.g., title and abstract screening, full-text review) included in the bibliometric analysis, including details of calibration exercises (e.g.,	5, Figure 1.

			number of researchers involved in process, roles in decision-making).	
**Data collection**		9	Describe the sources and methods of collecting bibliometric metadata ('data') including specifying which data was automatically versus manually extracted.	7, Figure 1.
**Data pre-processing**	Data loading and converting	10	Describe the process to load and convert the data.	7, Figure 1.
	Data cleaning strategy	11	Describe the process and strategy to clean the data, including software if used including the process to review data collected from database(s) to determine missing data and process to collect and input missing items.<br><br>Identify specific data items cleaned (e.g., relevant field tags).<br><br>Data cleaning decisions/actions (strategy) can be included as a table in an appendix.	7, Figure 1., Supplementary File 3, Supplementary File 4.
**Data analysis / synthesis**	Data analysis	14	List, define, and provide rationale for all bibliometric analysis techniques selected for analysis. Describe data analysis analytic parameters used for analyses and provide rationale. Analytic parameters can be identified in visualization legends and/or supplementary documents.	7, Supplementary File 3.
	Data visualizations	15	Describe the processes used to produce final visualizations (e.g., whether additional analysis was applied to software-generated visualizations).<br>Identify key parameters used in visualizations, if applicable, in the figure legends.<br>Provide detail on post work done to visualizations, if applicable, in the figure legends.	7, Figure 1., Supplementary File 3,
**Software Used**		13	Identify any software used throughout all processes (e.g., data pre-processing, analysis/visualization) and provide rationale. If multiple software applications were used, identify which processes each software was used for.	Figure 1.
**REPORTING**				
Characteristics of data set		16	Provide total counts of records returned for each database	Figure 1., Supplementary

Mapping the environmental co-benefits of reducing low-value care: A scoping review and bibliometric analysis
Parker et al., 2024

		searched, assessed for eligibility, and included in the bibliometric analysis, with reasons for exclusions at each stage. If the data set is small, a table can be provided in a supplementary file.	File 5, Supplementary File 6.
Flow diagram	17	Provide a flow diagram illustrating all processes in bibliometric analysis, including:<br>- selection of publications<br>- data collection<br>- data pre-processing<br>- data analysis / visualization<br>- data reporting (as applicable, identify which analyses are 'text-based' versus 'visualization-based')	Figure 1.
**DISCUSSION**			
Summary of data set	21	Summarize the main results link to the study objectives and research questions.	11 – 13
Limitations	22	Discuss any limitations of the bibliometric analysis process.	12
Conclusions	23	Provide a general interpretation of the results with respect to the study questions and research questions, as well as potential implications and/or next steps.	14
**FUNDING & DISCLOSURE OF INTEREST**			
Funding	24	Describe sources of funding and role of funders for the bibliometric analysis.	14
Disclosures of interest	25	Include disclosures of interest for all authors.	14

Adapted from: Tricco AC, Lillie E, Zarin W, O'Brien KK, Colquhoun H, Levac D, et al. PRISMA Extension for Scoping Reviews (PRISMA-ScR): Checklist and Explanation. Ann Intern Med;169:467–473. doi:10.7326/M18-0850
