# Supplementary material for: Mapping the Environmental Co-Benefits of Reducing Low-Value Care: A Scoping Review and Bibliometric Analysis
Source: Int J Environ Res Public Health. 2024 Jun 22;21(7):818. doi: 10.3390/ijerph21070818 (PMC11276457; doi:10.3390/ijerph21070818)
Supplement: Supplementary file 1 [file ijerph-21-00818-s001.zip › Supp File 3. Medline Search Strategy.pdf]

Supplementary File 3. Medline & Embase Search Strategy.

Embase Classic+Embase <1947 to 2023 July 1>

Ovid MEDLINE(R) ALL <1946 to July 1, 2023>

1 ("carbon footprint*" or "carbon emission*" or "climate change" or "net zero" or "climate risk" or "low carbon" or decarbon* or de-carbon* or "carbon performance" or co2e or "indirect carbon impact*" or "environmental impact" or "GHG emission*" or "environmental emission*" or "greenhouse gas emission*" or carbon-constrained or "environmentally sustainable" or "environmental sustainability").mp. [mp=ti, ab, hw, tn, ot, dm, mf, dv, kf, fx, dq, bt, nm, ox, px, rx, an, ui, sy]

2 (health* or healthcare or medicine* or medica* or "commissioned care" or hospital* or laborator*).mp. [mp=ti, ab, hw, tn, ot, dm, mf, dv, kf, fx, dq, bt, nm, ox, px, rx, an, ui, sy]

3 (overdiagnos* or "low value" or low-value or overtest* or over-test* or overtreat* or over-treat* or "choosing wisely" or "less is more" or "reducing waste" or overuse or "minimal benefit*" or de-implement* or deimplement* or deadopt* or de-adopt* or deprescrib* or de-prescrib* or unnecessary).af.

4 1 and 2 and 3

5 ("carbon footprint*" or "carbon emission*" or "climate change" or "net zero" or "climate risk" or "low carbon" or decarbon* or de-carbon* or "carbon performance" or co2e or "indirect carbon impact*" or "environmental impact" or "GHG emission* " or "environmental emission*" or "greenhouse gas emission*" or carbon-constrained or "environmentally sustainable" or "environmental sustainability").af.

6 2 and 3 and 5

7 remove duplicates from 6

8 limit 7 to humans

9 agricultur*.mp. [mp=ti, ab, hw, tn, ot, dm, mf, dv, kf, fx, dq, bt, nm, ox, px, rx, an, ui, sy]

10 7 not 9 244

11 (reduc* adj3 ("carbon footprint*" or "carbon emission*" or "climate change" or "net zero" or "climate risk" or "low carbon" or decarbon* or de-carbon* or "carbon performance" or co2e or "indirect carbon impact*" or "environmental impact" or "GHG emission*" or "environmental emission*" or "greenhouse gas emission*" or carbon-constrained or "environmentally sustainable" or "environmental sustainability")).ab. 10834

12 2 and 11 2406

13 limit 12 to humans 1392

14 agricultur*.mp. [mp=ti, ab, hw, tn, ot, dm, mf, dv, kf, fx, dq, bt, nm, ox, px, rx, an, ui, sy] 359912

15 13 not 14 1273

16 remove duplicates from 15 872

17 nonhuman.mp. [mp=ti, ab, hw, tn, ot, dm, mf, dv, kf, fx, dq, bt, nm, ox, px, rx, an, ui, sy] 7308827

18 16 not 17 814

19 limit 18 to yr="2019 -Current" 509

20 10 or 19 727

21 remove duplicates from 20 727

SCOPUS

Feb 1, 2023

1,121 document results

(TITLE-ABS-KEY (("carbon footprint*" OR "carbon emission*" OR "climate change" OR "net zero" OR "climate risk" OR "low carbon" OR decarbon* OR de-carbon* OR "carbon performance" OR co2e OR "indirect carbon impact*" OR "environmental impact" OR "GHG emission*" OR "environmental emission*" OR "greenhouse gas emission*" OR carbon-constrained OR "environmentally sustainable" OR "environmental sustainability") AND (health* OR healthcare OR medicine* OR medica* OR "commissioned care" OR hospital* OR laborator*))

AND ALL (overdiagnos* OR "low value" OR low-value OR overtest* OR over-test* OR overtreat* OR over-treat* OR "choosing wisely" OR "less is more" OR "reducing waste" OR overuse OR "minimal benefit*" OR de-implement* OR deimplement* OR deadopt* OR de-adopt* OR deprescrib* OR de-prescrib* OR unnecessary))

OR ((ALL ("carbon footprint*" OR "carbon emission*" OR "climate change" OR "net zero" OR "climate risk" OR "low carbon" OR decarbon* OR de-carbon* OR "carbon performance" OR co2e OR "indirect carbon impact*" OR "environmental impact" OR "GHG emission* " OR "environmental emission*" OR "greenhouse gas emission*" OR carbon-constrained OR "environmentally sustainable" OR "environmental sustainability")) AND (TITLE-ABS-KEY (health* OR healthcare OR medicine* OR medica* OR "commissioned care" OR hospital* OR laborator*) AND (overdiagnos* OR "low value" OR low-value OR overtest* OR over-test* OR overtreat* OR over-treat* OR "choosing wisely" OR "less is more" OR "reducing waste" OR overuse OR "minimal benefit*" OR de-implement* OR deimplement* OR deadopt* OR de-adopt* OR deprescrib* OR de-prescrib* OR unnecessary)))

AND (EXCLUDE (SUBJAREA , "AGRI") OR EXCLUDE (SUBJAREA , "ENGI") OR EXCLUDE (SUBJAREA , "EART") OR EXCLUDE (SUBJAREA , "COMP") OR EXCLUDE (SUBJAREA , "CHEM") OR EXCLUDE (

SUBJAREA , "CENG") OR EXCLUDE (SUBJAREA , "PHYS") OR EXCLUDE (SUBJAREA , "MATH") OR EXCLUDE (SUBJAREA , "VETE")) AND (EXCLUDE (EXACTKEYWORD , "Nonhuman") OR EXCLUDE (EXACTKEYWORD , "Animals") OR EXCLUDE (EXACTKEYWORD , "Animal") OR EXCLUDE (EXACTKEYWORD , "Agriculture") OR EXCLUDE (EXACTKEYWORD , "Soil") OR EXCLUDE (EXACTKEYWORD , "Chemistry") OR EXCLUDE (EXACTKEYWORD , "Particulate Matter") OR EXCLUDE (EXACTKEYWORD , "Geographic Distribution") OR EXCLUDE (EXACTKEYWORD , "Agricultural Worker") OR EXCLUDE (EXACTKEYWORD , "Malaria") OR EXCLUDE (EXACTKEYWORD , "Nitrogen") OR EXCLUDE (EXACTKEYWORD , "Spatiotemporal Analysis") OR EXCLUDE (EXACTKEYWORD , "Landfill") OR EXCLUDE (EXACTKEYWORD , "Agricultural Land") OR EXCLUDE (EXACTKEYWORD , "Fertilizer"))
