# Supplementary material for: Mapping the Environmental Co-Benefits of Reducing Low-Value Care: A Scoping Review and Bibliometric Analysis
Source: Int J Environ Res Public Health. 2024 Jun 22;21(7):818. doi: 10.3390/ijerph21070818 (PMC11276457; doi:10.3390/ijerph21070818)
Supplement: Supplementary file 1 [file ijerph-21-00818-s001.zip › Supp File 4_Study methods overview.pdf]

Mapping the environmental co-benefits of reducing low-value care: A scoping review and bibliometric analysis
Parker et al., 2024

Supplementary File 4. Study methods overview.

RQ	Fig	Analysis	Text Only	Viz?	Data Analyzed	Analytic Technique	Analytic Parameters Used	Analytic Software Used	Graphic Software Used
**Study Design Reporting**									
N/A	1.	**Adapted PRISMA Flow Diagram**	N/A	N/A		N/A	N/A	N/A	Adobe Illustrator
**Data Reporting & Visualization**									
**Scoping Review**									
How has knowledge production in this field evolved over time?	N/A	**Type of Publication**	X		Publication content	Content (to code data) / Thematic (to categorize type)	Publication-type counts, including yearly totals	Manual data analysis *(facilitated using Microsoft Excel)*	N/A
	2.	**Publications Over Time**		X	Bibliometric Metadata (Field tag: 'PY')	Publication	Yearly total publication counts	Manual data analysis *(facilitated using Microsoft Excel)*	N/A
What are the key and emerging area of focus in this field?	N/A	**Relative Co-benefits Focus**	X		Publication content	Content (to code data) / Thematic (to categorize focus)	Total counts comparing categories:<br>• publications (approximately) equally focused on environmental sustainability of healthcare and reducing LVC<br>• publications primarily focused on environmental sustainability of healthcare (and that acknowledge the benefits of reducing LVC)<br>• publications primarily focused on the health benefits of reducing LVC (and that acknowledge the environmental co-benefits of doing so)	Manual data analysis *(facilitated using Microsoft Excel)*	N/A
	3.	**Healthcare Focus**		X	Publication content	Content (to code data) / Thematic (to categorize focus)	Total counts comparing categories:<br>• Procedures<br>• System organization/design/evaluation<br>• Pharmaceuticals<br>• Care type/Setting	Manual data analysis *(facilitated using Microsoft Excel)*	Microsoft Excel *(base visualization);*<br>Adobe Illustrator *(post-work)*
	4.	**Environment / Sustainability Focus**		X	Publication content	Content (to code data) / Thematic (to categorize focus)	Total counts comparing categories:<br>• GHG emissions<br>• Pollution<br>• Resource use<br>• Waste management<br>• Supply chain & facility/service design<br>• Environmental stewardship<br>Total counts comparing within each category:<br>• Evidence / recommendation (if a study cited existing evidence or made a recommendation in an environmental sustainability category)	Manual data analysis *(facilitated using Microsoft Excel)*	Microsoft Excel *(base visualization);*<br>Adobe Illustrator *(post-work)*

							• Reported outcomes (if a study reported new study outcomes in an environmental sustainability category); empirical studies only (n = 13)		
	**Bibliometric Analysis**								
What are the prolific collaborations in this field?	5.	**Author Collaboration Network**		X	Bibliometric metadata (‘AU’)	Co-Authorship	**Biblioshiny analysis used:** ‘Collaboration Network’<br>**Field**: Authors<br>**Network layout**: Automatic (default)<br>**Clustering algorithm**: Walktrap (default)<br>**Normalization**: Association (default)<br>**Number of nodes**: 58 (top 10% of total authors)<br>**Repulsion force**: 0.1 (default)<br>**Remove isolated nodes**: Yes (default)<br>**Minimum number of edges**: 1 (default)<br><br>**Additional analysis completed by study authors:** healthcare focus of publications (from scoping review)	Biblioshiny;<br>Manual data analysis *(facilitated using Microsoft Excel)*	Biblioshiny *(base visualization);*<br>Adobe Illustrator *(post-work)*
	6.	**Country Collaboration Network**		X	Bibliometric metadata (‘C1’)	Co-Authorship	**Biblioshiny analysis used:** ‘Collaboration Network’<br>**Field**: Countries<br>**Network layout**: Automatic (default)<br>**Clustering algorithm**: Walktrap (default)<br>**Normalization**: Association (default)<br>**Number of nodes**: 200 (all countries in world, rounded)<br>**Repulsion force**: 0.1 (default)<br>**Remove isolated nodes**: Yes (default)<br>**Minimum number of edges**: 1 (default)<br><br>**Additional analysis completed by study authors:** number of publications generated by each network (identified number of publications produced by each country within the network; then, identified the specific publications produced by pairs of countries within each network)	Biblioshiny;<br>Manual data analysis *(facilitated using Microsoft Excel)*	Biblioshiny (base visualization);<br>Adobe Illustrator *(post-work)*
Who/what are the prolific authors, institutions, countries and publication sources in this field?	N/A	**Top Author Production Over Time**	X		Bibliometric metadata (‘AU’, ‘PY’)	Publication	**Biblioshiny analysis used:** ‘Authors’ Production over Time’<br>**Number of authors**: 19	Biblioshiny;<br>Manual data analysis *(facilitated using Microsoft Excel)*	N/A
	7.	**Top Institutions**		X	Bibliometric metadata (‘C1’)	Publication	**Biblioshiny analysis used:** ‘Most Relevant Affiliations’<br>**Affiliation Name Disambiguation**: No<br>**Number of Affiliations**: 16	Biblioshiny;<br>Manual data analysis	Biblioshiny *(base visualization);*<br>Adobe Illustrator *(post-work)*

							**Additional analysis completed by study authors:** institution country of origin; institution-type		
	N/A	**Total Number of Institutions**	X		Bibliometric metadata ('C1')	Publication	**'C1' > Edit column > Add column based on this column**<br>**'C1_v2' > Edit cells > Split multi-valued cells**<br>**'C1_v2' > Facet > Text facet**	OpenRefine	N/A
	N/A	**Top Publication Sources**	X		Bibliometric metadata ('SO')	Publication	**Biblioshiny analysis used:** 'Most Relevant Sources'<br>**Number of authors**: 6	Biblioshiny;<br>Manual data analysis	N/A
	N/A	**Total Number of Sources**	X		Bibliometric metadata ('SO')	Publication	**'SO' > Edit column > Add column based on this column**<br>**'SO_v2' > Edit cells > Split multi-valued cells**<br>**'SO_v2' > Facet > Text facet**	OpenRefine	N/A
