# Supplementary material for: Mapping the Environmental Co-Benefits of Reducing Low-Value Care: A Scoping Review and Bibliometric Analysis
Source: Int J Environ Res Public Health. 2024 Jun 22;21(7):818. doi: 10.3390/ijerph21070818 (PMC11276457; doi:10.3390/ijerph21070818)
Supplement: Supplementary file 1 [file ijerph-21-00818-s001.zip › Supp File 5_ Bibliometric analysis data cleaning actions.pdf]

Mapping the environmental co-benefits of reducing low-value care: A scoping review and bibliometric analysis
Parker et al., 2024

Supplementary File 5. Bibliometric analysis data cleaning actions

**Data-type (WoS field tag)**	**Cleaning action(s)**
author name ('AU')	• confirmed whether similar author names reflected the same or distinct authors<br>• ensured the consistent formatting of author names ('last name' 'first initial')
author institution / affiliation ('C1')	• retrieved and input 'missing' author affiliations<br>• ensured the consistent formatting of affiliates ('highest-level organization name', 'city', 'country')<br>• ensured distinct affiliates listed only once per publication<br>• ensured multiple instances of affiliates reflected affiliate headquarters or main campus location
publication source / journal ('SO')	• ensured the consistent formatting of sources ('highest-level source name').
