# Supplementary material for: Mapping the Environmental Co-Benefits of Reducing Low-Value Care: A Scoping Review and Bibliometric Analysis
Source: Int J Environ Res Public Health. 2024 Jun 22;21(7):818. doi: 10.3390/ijerph21070818 (PMC11276457; doi:10.3390/ijerph21070818)
Supplement: Supplementary file 1 [file ijerph-21-00818-s001.zip › Supp File 6_Included publications.pdf]

No.5	First Author	Title	Year	Journal	Reported Outcomes Analyzed (n = 13)
1	ABBASI, K	A SYSTEM RESET FOR THE CAMPAIGN AGAINST TOO MUCH MEDICINE	2022	BMJ-BRITISH MEDICAL JOURNAL	
2	ADEBISI, Y	BALANCING THE RISKS AND BENEFITS OF ANTIBIOTIC USE IN A GLOBALIZED WORLD: THE ETHICS OF ANTIMICROBIAL RESISTANCE	2023	GLOBALIZATION AND HEALTH	
3	AFANASJEVA, J	PHARMACISTS AS ENVIRONMENTAL STEWARDS: STRATEGIES FOR MINIMIZING AND MANAGING DRUG WASTE	2019	SUSTAINABLE CHEMISTRY AND PHARMACY	
4	AGBAFE, V	PRESCRIPTIONS FOR MITIGATING CLIMATE CHANGE-RELATED EXTERNALITIES IN CANCER CARE: A SURGEON'S PERSPECTIVE	2022	JOURNAL OF CLINICAL ONCOLOGY	
5	ARONSON, J	WHEN I USE A WORD .... TOO MUCH HEALTHCARE-OVERDETECTION	2022	BMJ-BRITISH MEDICAL JOURNAL	
6	AUNG, K	APPLYING AN ENVIRONMENTAL LENS TO VALUE-BASED CARE: A RESIDENT-DRIVEN PROJECT TO LIMIT LABORATORY TESTING ON AN INTERNAL MEDICAL WARD SERVICE	2021	THE JOURNAL OF CLIMATE CHANGE AND HEALTH	X
7	BADRICK, T	THE ROLE OF LABORATORIES IN REDUCING THE CARBON FOOTPRINT WASTE: REDUCE IT BEFORE IT REDUCES YOU	2022	AMERICAN JOURNAL OF CLINICAL PATHOLOGY	
8	BAILEY, C	CRISIS CARE: TACKLING THE CLIMATE AND ECOLOGICAL EMERGENCY	2021	BJPSYCH BULLETIN	
9	BARRATT, A	HIGH VALUE HEALTH CARE IS LOW CARBON HEALTH CARE CULLING LOW VALUE CARE WILL CUT HEALTH CARE CARBON EMISSIONS	2022	MEDICAL JOURNAL OF AUSTRALIA	
10	BARRATT, A	ENVIRONMENTAL IMPACT OF CARDIOVASCULAR HEALTHCARE	2023	OPEN HEART	
11	BARRATT, A	OVERDIAGNOSIS IS INCREASING THE CARBON FOOTPRINT OF HEALTHCARE	2021	BMJ-BRITISH MEDICAL JOURNAL	
12	BASU, P	ANTIBIOTIC RESISTANCE: THE IMPACT ON ENVIRONMENT AND SUSTAINABILITY	2013	INTERNATIONAL JOURNAL OF ENVIRONMENTAL SUSTAINABILITY	
13	BATCUP, C	BEHAVIOURAL CHANGE INTERVENTIONS ENCOURAGING CLINICIANS TO REDUCE CARBON EMISSIONS IN CLINICAL ACTIVITY: A SYSTEMATIC REVIEW	2023	BMC HEALTH SERVICES RESEARCH	
14	BEIN, T	THE CO2 FOOTPRINT OF INTENSIVE CARE MEDICINE-LET'S GO GREEN	2023	MEDIZINISCHE KLINIK-INTENSIVMEDIZIN UND NOTFALLMEDIZIN	
15	BERNSTEIN, A	PATIENT-CENTERED CLIMATE ACTION AND HEALTH EQUITY	2022	JAMA-JOURNAL OF THE AMERICAN MEDICAL ASSOCIATION	
16	BLACK, J	PLANNED DEPRESCRIBING TO PROTECT HEALTH SYSTEMS IN PANDEMICS AND OTHER DISASTERS: A SCOPING REVIEW	2022	CNS SPECTRUMS	

No.	First Author	Title	Year	Journal	Reported Outcomes Analyzed (n = 13)
17	BORN, K	REFRAMING RESOURCE STEWARDSHIP AND SUSTAINABILITY AS PROFESSIONALISM: WHAT CAN EFFORTS FOR A NET-ZERO HEALTH SYSTEM LEARN FROM CHOOSING WISELY CAMPAIGNS?	2020	HEALTHCARE PAPERS	
18	BORN, K	REDUCING HARM FROM OVERUSE OF HEALTHCARE	2022	BMJ-BRITISH MEDICAL JOURNAL	
19	BORN, K	CHOOSING WISELY AND THE CLIMATE CRISIS: A ROLE FOR CLINICIANS	2023	BMJ QUALITY & SAFETY	
20	BRADFORD, A	BUILDING CLINICAL PATHWAYS OF THE FUTURE THAT IMPROVE SAFETY AND REDUCE WASTE IN HEALTHCARE	2023	JOURNAL OF HOSPITAL MEDICINE	
21	BRETH-PETERSEN, M	HEALTH, FINANCIAL AND ENVIRONMENTAL IMPACTS OF UNNECESSARY VITAMIN D TESTING: A TRIPLE BOTTOM LINE ASSESSMENT ADAPTED FOR HEALTHCARE	2022	BMJ OPEN	X
22	BROWN, M	CLIMATE CHANGE AND RADIOLOGY: IMPETUS FOR CHANGE AND A TOOLKIT FOR ACTION	2023	RADIOLOGY	
23	CASULA, M	APPROPRIATENESS OF THE PRESCRIPTION AND USE OF MEDICINES: AN OLD CONCEPT BUT MORE RELEVANT THAN EVER	2023	INTERNATIONAL JOURNAL OF ENVIRONMENTAL RESEARCH AND PUBLIC HEALTH	
24	CHAKMA, M	INHALERS AND THE ENVIRONMENT: POLLUTION, PLASTICS AND POLICY	2022	PNEUMON	
25	CHARLESWORTH, K	NEW SOURCES OF VALUE FOR HEALTH AND CARE IN A CARBON-CONSTRAINED WORLD	2017	JOURNAL OF PUBLIC HEALTH	
26	CHARLESWORTH, K	ADDRESSING THE CARBON FOOTPRINT OF HEALTH ORGANISATIONS: EIGHT LESSONS FOR IMPLEMENTATION	2018	PUBLIC HEALTH RESEARCH & PRACTICE	
27	CONRY, J	CLIMATE CHANGE AND WOMEN'S HEALTH: TURNING LEADERSHIP INTO ACTION	2023	INTERNATIONAL JOURNAL OF GYNECOLOGY & OBSTETRICS	
28	CROOKS, M	IMPROVING ASTHMA CARE THROUGH IMPLEMENTATION OF THE SENTINEL PROGRAMME: FINDINGS FROM THE PILOT SITE	2023	ERJ OPEN RESEARCH	X
29	CROUCH, G	SUSTAINABILITY IN SURGERY: IMPLICATIONS FOR THE FUTURE	2022	ANZ JOURNAL OF SURGERY	
30	CUSSANS, A	INTERVENTIONS TO REDUCE THE ENVIRONMENTAL IMPACT OF MEDICINES: A UK PERSPECTIVE	2021	THE JOURNAL OF CLIMATE CHANGE AND HEALTH	
31	DAHLEN, H	SUPPORTING MIDWIFERY IS THE ANSWER TO THE WICKED PROBLEMS IN MATERNITY CARE	2022	LANCET GLOBAL HEALTH	
32	DAUGHTON, C	LOWER-DOSE PRESCRIBING: MINIMIZING "SIDE EFFECTS" OF PHARMACEUTICALS ON SOCIETY AND THE ENVIRONMENT	2013	SCIENCE OF THE TOTAL ENVIRONMENT	
33	DAVIES, B	GERIATRIC MEDICINE IN THE ERA OF CLIMATE CHANGE	2022	AGE AND AGEING	
34	DAVIES, J	IMPLEMENTATION APPROACHES TO IMPROVE ENVIRONMENTAL SUSTAINABILITY IN OPERATING THEATRES: A SYSTEMATIC REVIEW	2023	BRITISH JOURNAL OF ANAESTHESIA	

No.	First Author	Title	Year	Journal	Reported Outcomes Analyzed (n = 13)
35	DAVIES, J	OPERATION CLEAN UP: A MODEL FOR ECO-LEADERSHIP AND SUSTAINABILITY IMPLEMENTATION	2023	ANAESTHESIA AND INTENSIVE CARE	X
36	DE FREITAS, L	CARIBBEAN HEALTH PROFESSIONAL VIEWS ON CLIMATE CHANGE AND HEALTH	2023	THE JOURNAL OF CLIMATE CHANGE AND HEALTH	
37	DE LAS VECILLAS	LANDSCAPE OF SHORT-ACTING BETA-AGONISTS (SABA) OVERUSE IN EUROPE	2022	CLINICAL AND EXPERIMENTAL ALLERGY	
38	DICKSON, K	PERSPECTIVES ON CLIMATE CHANGE: CAN HAND SURGERY GO CARBON NEUTRAL?	2023	JOURNAL OF HAND SURGERY-EUROPEAN VOLUME	
39	DONNELLY, M	SUSTAINABILITY AND SLEEP MANAGEMENT IN PSYCHIATRIC WARDS	2020	BRITISH JOURNAL OF MEDICAL PRACTITIONERS	
40	DOSSETT, L	REDUCING UNNECESSARY PREOPERATIVE TESTING	2022	BMJ-BRITISH MEDICAL JOURNAL	
41	DUAN, X	STATUS OF PHLEBOTOMY TUBE UTILIZATION AT A MAJOR MEDICAL CENTER. ARE WE USUING TOO MANY PHLEBOTOMY TUBES?	2023	HELIYON	
42	ECKELMAN, M	HEALTH CARE POLLUTION AND PUBLIC HEALTH DAMAGE IN THE UNITED STATES: AN UPDATE	2020	HEALTH AFFAIRS	
43	EVANS, A	INCORPORATING 'GREEN PODIATRY' INTO YOUR CLINIC, AND INTO YOUR LIFE	2022	JOURNAL OF FOOT AND ANKLE RESEARCH	
44	FAN, B	REDUCING OUR CARBON FOOTPRINT IN THE HAEMATOLOGY LABORATORY: A SHARED RESPONSIBILITY	2023	INTERNATIONAL JOURNAL OF LABORATORY HEMATOLOGY	
45	FARRELL, B	REDUCE UNNECESSARY USE OF PROTON PUMP INHIBITORS	2022	BMJ-BRITISH MEDICAL JOURNAL	
46	FURLAN, L	CHOOSING WISELY IN CLINICAL PRACTICE: EMBRACING CRITICAL THINKING, STRIVING FOR SAFER CARE	2022	JOURNAL OF INTERNAL MEDICINE	X
47	FURLAN, L	THE ENVIRONMENTAL COST OF UNWARRANTED VARIATION IN THE USE OF MAGNETIC RESONANCE IMAGING AND COMPUTED TOMOGRAPHY SCANS	2023	EUROPEAN JOURNAL OF INTERNAL MEDICINE	
48	GARCIA-BLASCO, C	PATIENT'S PREFERENCES AND SHARED DECISION-MAKING: CONSIDERING ENVIRONMENTAL ASPECTS IN HEAD LICE TREATMENT	2023	SUSTAINABLE CHEMISTRY AND PHARMACY	
49	GEORGE, A	THE VALUE OF LESS: IMPROVING PATIENT CARE BY REDUCING UNNECESSARY PROCEDURES	2022	NEJM CATALYST INNOVATIONS IN CARE DELIVERY	
50	GORDON, I	LIFE CYCLE GREENHOUSE GAS EMISSIONS OF GASTROINTESTINAL BIOPSIES IN A SURGICAL PATHOLOGY LABORATORY	2021	AMERICAN JOURNAL OF CLINICAL PATHOLOGY	

No.	First Author	Title	Year	Journal	Reported Outcomes Analyzed (n = 13)
51	GREAVES, R	KEY QUESTIONS ABOUT THE FUTURE OF LABORATORY MEDICINE IN THE NEXT DECADE OF THE 21ST CENTURY: A REPORT FROM THE IFCC-EMERGING TECHNOLOGIES DIVISION	2019	CLINICA CHIMICA ACTA	
52	GRIMES, L	REDUCING UNNECESSARY BLOOD TESTS ON A HAEMATOLOGY WARD AT SALISBURY NHS FOUNDATION TRUST	2022	BRITISH JOURNAL OF HAEMATOLOGY	
53	HALE, I	FAMILY DOCTORS WELL SUITED TO BEING CLIMATE LEADERS	2023	CANADIAN FAMILY PHYSICIAN	
54	HALE, I	PRACTISING ENVIRONMENTALLY SUSTAINABLE HEALTH CARE EVERY DAY	2023	BRITISH COLUMBIA MEDICAL JOURNAL	
55	HEATH, I	BOOST FOR SUSTAINABLE HEALTHCARE	2020	BMJ-BRITISH MEDICAL JOURNAL	
56	HENSHER, M	INCORPORATING ENVIRONMENTAL IMPACTS INTO THE ECONOMIC EVALUATION OF HEALTH CARE SYSTEMS: PERSPECTIVES FROM ECOLOGICAL ECONOMICS	2020	RESOURCES CONSERVATION AND RECYCLING	
57	HENSHER, M	HEALTH CARE, OVERCONSUMPTION AND UNECONOMIC GROWTH: A CONCEPTUAL FRAMEWORK	2020	SOCIAL SCIENCE & MEDICINE	
58	HENSHER, M	HEALTH CARE SUSTAINABILITY METRICS: BUILDING A SAFER, LOW-CARBON HEALTH SYSTEM	2020	HEALTH AFFAIRS	
59	HENSHER, M	"TOO MUCH MEDICINE": INSIGHTS AND EXPLANATIONS FROM ECONOMIC THEORY AND RESEARCH	2017	SOCIAL SCIENCE & MEDICINE	
60	HENSHER, M	CAN HEALTHCARE ADAPT TO A WORLD OF TIGHTENING ECOLOGICAL CONSTRAINTS? CHALLENGES ON THE ROAD TO A POST-GROWTH FUTURE	2020	BMJ-BRITISH MEDICAL JOURNAL	
61	JOHANSSON, M	THE RECOVERY-A PODCAST ABOUT ACTION FOR SUSTAINABLE HEALTHCARE	2021	BMJ-BRITISH MEDICAL JOURNAL	
62	KAMPMAN, J	RANDOMIZED CONTROLLED TRIALS INSUFFICIENTLY FOCUS ON REDUCING MEDICAL OVERUSE	2023	EUROPEAN JOURNAL OF EPIDEMIOLOGY	
63	KAMPMAN, J	ANAESTHESIA AND ENVIRONMENT: IMPACT OF A GREEN ANAESTHESIA ON ECONOMICS	2023	CURRENT OPINION IN ANESTHESIOLOGY	
64	KIANG, K	DELIVERING ENVIRONMENTAL SUSTAINABILITY IN HEALTHCARE FOR FUTURE GENERATIONS: TIME TO CLEAN UP OUR OWN CUBBY HOUSE	2021	JOURNAL OF PAEDIATRICS AND CHILD HEALTH	
65	KOPPARAM, R	THE ENVIRONMENTAL IMPACT OF UNNECESSARY IMAGING: WHY LESS IS MORE	2023	EUROPEAN JOURNAL OF INTERNAL MEDICINE	
66	KRAEMER, S	ANTIBIOTIC POLLUTION IN THE ENVIRONMENT: FROM MICROBIAL ECOLOGY TO PUBLIC POLICY	2019	MICROORGANISMS	
67	LEAPMAN, M	ENVIRONMENTAL IMPACT OF PROSTATE MAGNETIC RESONANCE IMAGING AND TRANSRECTAL ULTRASOUND GUIDED PROSTATE BIOPSY	2023	EUROPEAN UROLOGY	X
68	LEE, V	DECARBONIZING HEALTH CARE: ENGAGING LEADERS IN CHANGE	2023	NEJM CATALYST INNOVATIONS IN CARE DELIVERY	
69	LEGRAND, J	SUSTAINABLE DEVELOPMENT IN GENERAL PRACTICE	2022	FAMILY PRACTICE	

No.	First Author	Title	Year	Journal	Reported Outcomes Analyzed (n = 13)
70	LI, C	THE ROLE OF MENTAL HEALTH PROFESSIONALS IN THE CLIMATE CRISIS: AN URGENT CALL TO ACTION	2022	INTERNATIONAL REVIEW OF PSYCHIATRY	
71	LLOR, C	DEPRESCRIBING IN OLD PEOPLE: ONLY FOR CHRONIC MEDICATION?	2022	ATENCION PRIMARIA	
72	LOPES, R	INHALATIONAL ANAESTHETICS, OZONE DEPLETION, AND GREENHOUSE WARMING: THE BASICS AND STATUS OF OUR EFFORTS IN ENVIRONMENTAL MITIGATION	2021	CURRENT OPINION IN ANESTHESIOLOGY	
73	MADDEN, D	ENVIRONMENTALLY SUSTAINABLE HEALTH CARE: NOW IS THE TIME FOR ACTION	2020	MEDICAL JOURNAL OF AUSTRALIA	
74	MARRAULD, L	CARING FOR PATIENTS WHILE CARING FOR THE PLANET: THE CARBON FOOTPRINT OF THE FRENCH HEALTHCARE SYSTEMS AND LEVERS FOR ITS REDUCTION	2023	MEDECINE DES MALADIES METABOLIQUES	
75	MCALISTER, S	THE CARBON FOOTPRINT OF PATHOLOGY TESTING	2020	MEDICAL JOURNAL OF AUSTRALIA	X
76	MCALISTER, S	AN LCA OF HOSPITAL PATHOLOGY TESTING	2021	INTERNATIONAL JOURNAL OF LIFE CYCLE ASSESSMENT	
77	MCALISTER, S	THE CARBON FOOTPRINT OF HOSPITAL DIAGNOSTIC IMAGING IN AUSTRALIA	2022	INTERNATIONAL JOURNAL OF LIFE CYCLE ASSESSMENT	
78	MCALISTER, S	CARBON EMISSIONS AND HOSPITAL PATHOLOGY STEWARDSHIP: A RETROSPECTIVE COHORT ANALYSIS	2023	INTERNAL MEDICINE JOURNAL	X
79	MCGAIN, F	WHY BE SUSTAINABLE? THE AUSTRALIAN AND NEW ZEALAND COLLEGE OF ANAESTHETISTS PROFESSIONAL DOCUMENT PS64: STATEMENT ON ENVIRONMENTAL SUSTAINABILITY IN ANAESTHESIA AND PAIN MEDICINE PRACTICE AND ITS ACCOMPANYING BACKGROUND PAPER	2019	ANAESTHESIA AND INTENSIVE CARE	
80	MCGAIN, F	ENVIRONMENTAL SUSTAINABILITY IN ANAESTHESIA AND CRITICAL CARE	2020	BRITISH JOURNAL OF ANAESTHESIA	
81	MCGAIN, F	ENVIRONMENTAL SUSTAINABILITY IN HOSPITALS - A SYSTEMATIC REVIEW AND RESEARCH AGENDA	2014	JOURNAL OF HEALTH SERVICES RESEARCH & POLICY	
82	MCNALLY, S	LEAN PATHWAYS IN ORTHOPAEDICS: MULTIPLE WINS FOR SUSTAINABILITY	2022	JOURNAL OF CLINICAL ORTHOPAEDICS AND TRAUMA	
83	MICHAEL, C	THE ANTIMICROBIAL RESISTANCE CRISIS: CAUSES, CONSEQUENCES, AND MANAGEMENT	2014	FRONTIERS IN PUBLIC HEALTH	
84	MILOVAC, T	PHARMACEUTICALS IN THE WATER: THE NEED FOR ENVIRONMENTAL BIOETHICS	2023	JOURNAL OF MEDICAL HUMANITIES	

No.	First Author	Title	Year	Journal	Reported Outcomes Analyzed (n = 13)
85	MINOGUE, V	REDUCING WASTE IN THE NHS: AN OVERVIEW OF THE LITERATURE AND CHALLENGES FOR THE NURSING PROFESSION	2016	NURSING MANAGEMENT	
86	MIR, F	TACKLING OVERPRESCRIBING: LONG OVERDUE WITH A LOT TO DO	2021	BMJ-BRITISH MEDICAL JOURNAL	
87	MOLOO, H	PLANETARY HEALTH CARE FOR COLORECTAL SURGEONS	2022	DISEASES OF THE COLON & RECTUM	
88	MORRIS, K	MAKING PRACTICAL PROGRESS ON CLIMATE CHANGE IN HEALTHCARE: SEIZING OPPORTUNITIES	2020	HEALTHCARE PAPERS	
89	MORTIMER, F	SUSTAINABILITY IN QUALITY IMPROVEMENT: REDEFINING VALUE	2018	FUTURE HEALTHCARE JOURNAL	
90	NELSON, A	ENVIRONMENTAL PAIN APPROACH (EPA): SUSTAINABILITY IN CHRONIC PAIN PRACTICE	2023	PAIN MEDICINE	
91	PALM, V	SUSTAINABILITY AND CLIMATE PROTECTION IN RADIOLOGY - AN OVERVIEW	2023	ROFO	
92	PARK, SB	GASTROINTESTINAL ENDOSCOPY’S CARBON FOOTPRINT	2023	CLINICAL ENDOSCOPY	
93	PAVLI, A	WHAT CAN WE LEARN FROM AUSTRALIAN GENERAL PRACTICES TAKING STEPS TO BE MORE ENVIRONMENTALLY SUSTAINABLE? A QUALITATIVE STUDY	2023	FAMILY PRACTICE	
94	PENCHEON, D	MAKING HEALTHCARE AND HEALTH SYSTEMS NET ZERO	2020	BMJ-BRITISH MEDICAL JOURNAL	
95	PERNIGOTTI, D	REDUCING CARBON FOOTPRINT OF INHALERS: ANALYSIS OF CLIMATE AND CLINICAL IMPLICATIONS OF DIFFERENT SCENARIOS IN FIVE EUROPEAN COUNTRIES	2021	BMJ OPEN RESPIRATORY RESEARCH	
96	PICANO, E	ENVIRONMENTAL SUSTAINABILITY OF MEDICAL IMAGING	2021	ACTA CARDIOLOGICA	
97	PICANO, E	CLIMATE CHANGE, CARBON DIOXIDE EMISSIONS, AND MEDICAL IMAGING CONTRIBUTION	2023	JOURNAL OF CLINICAL MEDICINE	
98	PRAPHARSAVAT, R	RAISING AWARENESS OF ANTIMICROBIAL RESISTANCE: DEVELOPMENT OF AN 'ANTIBIOTIC FOOTPRINT CALCULATOR'	2023	JOURNAL OF ANTIMICROBIAL CHEMOTHERAPY	
99	PRASAD, P	ENVIRONMENTAL FOOTPRINT OF REGULAR AND INTENSIVE INPATIENT CARE IN A LARGE US HOSPITAL	2022	INTERNATIONAL JOURNAL OF LIFE CYCLE ASSESSMENT	
100	REDVERS, N	PHYSICIANS' VIEWS OF PATIENT-PLANETARY HEALTH CO-BENEFIT PRESCRIBING: A MIXED METHODS SYSTEMATIC REVIEW	2023	THE LANCET PLANETARY HEALTH	
101	REGAN, W	SUSTAINABLE APPROACH TO REDUCING UNNECESSARY COMBINED BIOCHEMISTRY TESTS ON A PAEDIATRIC CARDIOLOGY WARD	2018	BMJ OPEN QUALITY	X
102	RICHIE, C	ENVIRONMENTAL SUSTAINABILITY AND THE CARBON EMISSIONS OF PHARMACEUTICALS	2022	JOURNAL OF MEDICAL ETHICS	
103	RICHIE, C	CLIMATE CHANGE AND THE PRESCRIPTION PAD	2023	LANCET	

No.	First Author	Title	Year	Journal	Reported Outcomes Analyzed (n = 13)
104	RODRIGUEZ DE SANTIAGO, E	REDUCING THE ENVIRONMENTAL FOOTPRINT OF GASTROINTESTINAL ENDOSCOPY: EUROPEAN SOCIETY OF GASTROINTESTINAL ENDOSCOPY (ESGE) AND EUROPEAN SOCIETY OF GASTROENTEROLOGY AND ENDOSCOPY NURSES AND ASSOCIATES (ESGENA) POSITION STATEMENT	2022	LANCET	
105	RODRIGUEZ-JIMENEZ, L	THE CARBON FOOTPRINT OF HEALTHCARE SETTINGS: A SYSTEMATIC REVIEW	2023	JOURNAL OF ADVANCED NURSING	
106	SAHA, S	SUSTAINABLE TRANSFORMATION OF PRACTICES AND OUTPATIENT CLINICS	2023	DERMATOLOGIE	
107	SCHENK, E	NURSING WASTE REDUCTION FOR A HEALTHY ENVIRONMENT	2023	JOURNAL OF RADIOLOGY NURSING	
108	SEBASTIAN, S	GREEN ENDOSCOPY: BRITISH SOCIETY OF GASTROENTEROLOGY (BSG), JOINT ACCREDITATION GROUP (JAG) AND CENTRE FOR SUSTAINABLE HEALTH (CSH) JOINT CONSENSUS ON PRACTICAL MEASURES FOR ENVIRONMENTAL SUSTAINABILITY IN ENDOSCOPY	2023	GUT	
109	SELVAM, R	REDEFINING THE ROLE OF ROUTINE POSTOPERATIVE BLOODWORK FOLLOWING UNCOMPLICATED BARIATRIC SURGERY	2023	SURGICAL ENDOSCOPY AND OTHER INTERVENTIONAL TECHNIQUES	X
110	SERGEANT, M	WHAT HEALTHCARE LEADERSHIP CAN DO IN A CLIMATE CRISIS	2023	HEALTHCARE MANAGEMENT FORUM	
111	SERGEANT, M	IDENTIFYING OPPORTUNITIES FOR GREENHOUSE GAS REDUCTIONS AND COST SAVINGS IN HOSPITALS: A KNOWLEDGE TRANSLATION TREE	2022	HEALTHCARE QUARTERLY	
112	SHERMAN, J	SCALING UP INHALED ANESTHETIC PRACTICE IMPROVEMENT: THE ROLE OF ENVIRONMENTAL SUSTAINABILITY METRICS	2019	ANESTHESIA AND ANALGESIA	
113	SHERMAN, J	NET ZERO HEALTHCARE: A CALL FOR CLINICIAN ACTION	2021	BMJ-BRITISH MEDICAL JOURNAL	
114	SHERMAN, J	BRINGING ENVIRONMENTAL SUSTAINABILITY INTO THE QUALITY AGENDA: TIME TO ACT ON REDUCING HEALTH CARE POLLUTION AND WASTE	2023	JOINT COMMISSION JOURNAL ON QUALITY AND PATIENT SAFETY	
115	SHERMAN, J	THE GREEN PRINT: ADVANCEMENT OF ENVIRONMENTAL SUSTAINABILITY IN HEALTHCARE	2020	RESOURCES CONSERVATION AND RECYCLING	
116	SIGNORINI, S	LESS IS MORE: AN ECOLOGICAL AND ECONOMIC POINT OF VIEW ON APPROPRIATE USE OF LAB TESTING FOR COVID PATIENTS	2021	BIOANALYSIS	
117	SITTIG, D	I-CLIMATE: A "CLINICAL CLIMATE INFORMATICS" ACTION FRAMEWORK TO REDUCE ENVIRONMENTAL POLLUTION FROM HEALTHCARE	2022	JOURNAL OF THE AMERICAN MEDICAL INFORMATICS ASSOCIATION	

No.	First Author	Title	Year	Journal	Reported Outcomes Analyzed (n = 13)
118	SKOWNO, J	LIGHTING A CANDLE, OR CURSING THE DARKNESS? DELIVERING A CLIMATE FRIENDLY ANAESTHETIC	2021	JOURNAL OF PAEDIATRICS AND CHILD HEALTH	
119	SMITH, CL	WE CAN'T MITIGATE WHAT WE DON'T MONITOR: USING INFORMATICS TO MEASURE AND IMPROVE HEALTHCARE SYSTEMS' CLIMATE IMPACT AND ENVIRONMENTAL FOOTPRINT	2022	JOURNAL OF THE AMERICAN MEDICAL INFORMATICS ASSOCIATION	
120	SPOYALO, K	PATIENT, HOSPITAL AND ENVIRONMENTAL COSTS OF UNNECESSARY BLOODWORK: CAPTURING THE TRIPLE BOTTOM LINE OF INAPPROPRIATE CARE IN GENERAL SURGERY PATIENTS	2023	BMJ OPEN QUALITY	X
121	STUART, N	ENVIRONMENTAL SUSTAINABILITY MUST BE ON OUR AGENDA FOR HEALTHCARE	2020	HEALTHCARE PAPERS	
122	SZIRT, R	ENVIRONMENTAL SUSTAINABILITY IN THE CARDIAC CATHETER LABORATORY	2023	HEART LUNG AND CIRCULATION	
123	TALLEY, N	HEALTH AND CLIMATE CHANGE MJA-LANCET COUNTDOWN REPORT: AUSTRALIA GETS ANOTHER FAILING GRADE IN 2020 BUT SHOWS SIGNS OF PROGRESS	2021	LANCET	
124	TARRANT, C	OPTIMIZING ANTIBIOTIC PRESCRIBING: COLLECTIVE APPROACHES TO MANAGING A COMMON-POOL RESOURCE	2019	CLINICAL MICROBIOLOGY AND INFECTION	
125	TEN HAVE, P	HEALTH TECHNOLOGY ASSESSMENT LIFE CYCLE APPROACH IN ASTHMA CARE	2022	INTERNATIONAL JOURNAL OF TECHNOLOGY ASSESSMENT IN HEALTH CARE	
126	THIEL, C	CARBON EMISSIONS FROM OVERUSE OF US HEALTH CARE: MEDICAL AND ETHICAL PROBLEMS	2022	HASTINGS CENTER REPORT	
127	THOMAS, F	MEDICINE 'MISUSE': IMPLICATIONS FOR HEALTH AND ENVIRONMENTAL SUSTAINABILITY	2015	SOCIAL SCIENCE & MEDICINE	
128	TOMSON, C	REDUCING THE CARBON FOOTPRINT OF HOSPITAL-BASED CARE	2015	FUTURE HOSPITAL JOURNAL	
129	TRENT, L	CREATE INTENSIVE CARE GREEN TEAMS, THERE IS NO TIME TO WASTE	2023	INTENSIVE CARE MEDICINE	
130	TWIGG, A	LOCAL VARIATION IN LOW CARBON FOOTPRINT INHALERS IN PRE-COVID PANDEMIC PRIMARY CARE PRESCRIBING GUIDELINES FOR ADULT ASTHMA IN ENGLAND AND ITS POTENTIAL IMPACT	2022	BRITISH JOURNAL OF CLINICAL PHARMACOLOGY	X
131	UKUHOR, H	THE INTERRELATIONSHIPS BETWEEN ANTIMICROBIAL RESISTANCE, COVID-19, PAST, AND FUTURE PANDEMICS	2021	JOURNAL OF INFECTION AND PUBLIC HEALTH	
132	VALDIVIESO, D	WHY CLIMATE ACTIVISTS SHOULD CARE ABOUT HEALTHCARE WASTE AND OVERUS	2022	THE JOURNAL OF CLIMATE CHANGE AND HEALTH	
133	VAN EGMOND, P	FROM BIG HANDS TO GREEN FINGERS: IT IS TIME FOR A CHANGE	2023	JOURNAL OF ISAKOS	
134	WALPOLE, S	IMPROVING ANTIMICROBIAL USE TO PROTECT THE ENVIRONMENT: WHAT IS THE ROLE OF INFECTION SPECIALISTS?	2023	ANTIBIOTICS-BASEL	

No.	First Author	Title	Year	Journal	Reported Outcomes Analyzed (n = 13)
135	WALSH, O	EVERYONE'S A WINNER IF WE TEST LESS: THE CODA ACTION PLAN	2022	AUSTRALIAN HEALTH REVIEW	
136	WANG, E	ENVIRONMENTAL EMISSIONS REDUCTION OF A PREOPERATIVE EVALUATION CENTER UTILIZING TELEHEALTH SCREENING AND STANDARDIZED PREOPERATIVE TESTING GUIDELINES	2021	RESOURCES CONSERVATION AND RECYCLING	X
137	WHITE, S	PRINCIPLES OF ENVIRONMENTALLY-SUSTAINABLE ANAESTHESIA: A GLOBAL CONSENSUS STATEMENT FROM THE WORLD FEDERATION OF SOCIETIES OF ANAESTHESIOLOGISTS	2022	ANAESTHESIA	
138	WIGAND, M	CLIMATE CHANGE, POLLUTION, DEFORESTATION, AND MENTAL HEALTH: RESEARCH TRENDS, GAPS, AND ETHICAL CONSIDERATIONS	2022	GEOHEALTH	
139	WILKINSON, A	SUSTAINABILITY IN INHALED DRUG DELIVERY	2020	PHARMACEUTICAL MEDICINE	
140	WILKINSON, A	ENVIRONMENTAL SUSTAINABILITY IN RESPIRATORY CARE: AN OVERVIEW OF THE HEALTHCARE-BASED ENVIRONMENTAL COST OF TREATMENT (CARBON) PROGRAMME	2022	ADVANCES IN THERAPY	
141	WILKINSON, A	AN ASSESSMENT OF SHORT-ACTING B2-AGONIST (SABA) USE AND SUBSEQUENT GREENHOUSE GAS (GHG) EMISSIONS IN FIVE EUROPEAN COUNTRIES AND THE CONSEQUENCES OF THEIR POTENTIAL OVERUSE FOR ASTHMA IN THE UK	2021	THORAX	
142	WOOLEN, S	RADIOLOGY ENVIRONMENTAL IMPACT: WHAT IS KNOWN AND HOW CAN WE IMPROVE?	2023	ACADEMIC RADIOLOGY	
143	XIE, E	LEVERAGING OUR STRENGTHS TO ACHIEVE SUSTAINABLE HEALTHCARE	2020	HEALTHCARE PAPERS	
144	YIN, R	SUSTAINABLE GENERAL PRACTICE	2023	AUSTRALIAN JOURNAL OF GENERAL PRACTICE	
145	YUSUF, E	THE UNINTENDED CONTRIBUTION OF CLINICAL MICROBIOLOGY LABORATORIES TO CLIMATE CHANGE AND MITIGATION STRATEGIES: A COMBINATION OF DESCRIPTIVE STUDY, SHORT SURVEY, LITERATURE REVIEW AND OPINION	2022	CLINICAL MICROBIOLOGY AND INFECTION	
